# Supplementary material for: Trait-trait relationships and tradeoffs vary with genome size in prokaryotes
Source: Front Microbiol. 2022 Oct 21;13:985216. doi: 10.3389/fmicb.2022.985216 (PMC9634001; doi:10.3389/fmicb.2022.985216)
Supplement: Supplementary file 1 [file Data_Sheet_1.pdf]

Supplementary file

Material and Methods

Genomic traits We determined 11 genomic traits of 17,856 genomes integrated as reference database in the PICRUSt2 software (Douglas et al., 2020). The PICRUSt2 software was developed to predict genome information from 16s rRNA phylotypes of uncultured prokaryotes from close relatives with available complete genome information and genome information of the reference genomes is available via the JGI/IMG database (https://img.jgi.doe.gov/, Chen et al., 2021; Mukherjee et al., 2021). Some of the genomes present in the original PICRUSt2 reference database had meanwhile been replaced or removed. In the case of replaced genomes we indicate the new IMG genome ID in the column IMG.Genome.ID of Table S1, while in the column PICRUSt.ID the former IMG genome ID is given. Douglas and colleagues furthermore present averaged information of several genomes, if these contained identical 16s rRNA genes (picrust.ID = *-cluster). The trait data used in this study refer exclusively to the genomes indicated in column IMG.Genome.ID of Table S1.

Trait values for the GC content (%GC), genome size and the percentage of horizontally acquired genes (%HTG) were directly extracted from the genome statistics information provided via the JGI/IMG database. Gene richness was computed as the number of unique gene orthologs given in the *.cog.tab.txt file that is available for individual genomes via the JGI/IMG database (for instance 2593339298.cog.tab.txt for the genome with the IMG.Genome.ID 2593339298). Gene duplication was computed as the average number of entries in the *.cog.tab.txt file encoding one unique gene ortholog. To identify genes encoding transcription factors we used the DBD database (Wilson et al., 2008) that lists putative entries from the PFAM database (https://pfam.xfam.org/, Mistry et al., 2021) encoding transcription factors. Matches of DBD transcription factors given in the *.pfam.tab.txt file that is available for individual genomes via the JGI/IMG database were determined. To obtain the percentage of genes encoding transcription factors (%TF) within a genome, we divided the number of genes identified as transcription factors by the number of total genes provided via the JGI/IMG database (see values for Gene.count in Table S1). The number of prophages in each genome was determined via the VirSorter software (Roux et al., 2015) using the JGI/IMG *.genes.fna file as input file. We considered VirSorter predictions of the category 1 (sure) and 2 (somewhat sure) as prophages for downstream analyses. The codon usage bias (CUB) was determined via the parameter F as detailed by Viera Silva (2010) or as estimated maximal growth rate using the gRodon R package (Weissman et al., 2021). In both cases we used gene sequences available via the JGI/IMG *.genes.fna file and among these we considered genes annotated as ribosomal proteins (based on gene annotations in the *.cog.tab.txt file) as highly expressed genes.

The number of 16s rRNA gene operons (RRN) is given in genome statistics information provided via the JGI/IMG database. However, high RRN variances among closely related genomes indicate that JGI/IMG provided RRN values that were obtained from assembled genome sequence data may not be fully reliable. A possible explanation for this observation is that the 16s rRNA gene is highly conserved and (nearly) identical copies of this gene within a genome may collapse into a single 16s gene sequence during the assembly process and thereby result in an underestimation of the true RRN. We therefore extrapolated RRN values averaged at the NCBI genus level from the

rrnDB (Stoddard et al., 2015) via matches at the NCBI genus level of JGI/IMG genome entries from this study. The standard variation of rrnDB RRN values among strains within the same genus was in the majority of cases <1 (Figure S4 B) and we believed the extrapolation of RRN at the genus level to be sufficiently robust for the purpose of our study. RRN values from the rrnDB are also derived after genome assembly, but are manually curated. We further compared both, JGI/IMG RRN values and values extrapolated from the rrnDB against RRN values that were estimated from raw read data. Raw read-based RRN estimates are not subjected to any assembly dependent bias and were obtained by relating the number of reads encoding the 16s rRNA gene to the number of reads coding for obligatory single copy genes in a genome (Biers et al., 2009). This was possible for ~1500 genomes for which we could extract SRR runids from the NCBI Bioproject Accession number that is available for some of the JGI/IMG genomes (Table S1). We hereby excluded duplicate NCBI Bioproject Accessions from Table S1 because this may indicate that several genomes have been sequenced under a single NCBI Bioproject Accession. Also metagenome assembled genomes were excluded because in this case reads from a single genome cannot be separated out from the read file. The extracted SRR runids were used to download raw read files. To speed up the downstream analyses, we used only the reverse run file from paired-end reads and all read files were subsampled to maximal 2.000.000 reads. Read files ending with *consensus.fastq.gz or *subreads.fastq.gz were excluded, as these files result from sequencing methods producing long reads that may include more than one gene. We used the SortMeRNA software (Kopylova et al., 2012) to identify reads encoding the 16s rRNA gene. Subsequently we counted the number of 16s encoding reads and all reads and determined the average read length. The number of sequenced genome equivalents that indicates the sequence depth in relation to genome size was determined via the MicrobeCensus software (Nayfach and Pollard, 2015). The MicrobeCensus software output includes beside the number of sequenced genome equivalents also a read-based estimate of genome size that is computed from the ratio of all reads and reads encoding obligatory single copy genes. The read-based RRN was estimated by normalizing the number of reads encoding the 16s rRNA gene by read lengths relative to the lengths of the total 16s rRNA gene (in E. coli: 1541 base pairs) and divide the resulting value through the number of sequenced genome equivalents.

A scatterplot of assembly-based JGI/IMG versus rrnDB extrapolated RRN values (Figures S4 A) exhibited that JGI/IMG were often lower than the manually curated but still assembly-based rrnDB values. This confirmed our assumption that non-curated JGI/IMG values tend to underestimated the true RRN value. Correlations of JGI/IMG and rrnDB extrapolated RRN counts against read-based RRN estimates, further corroborated that rrnDB extrapolated RRN counts were less biased (Figure S5 A,B). However, a correlation of read-based genome size estimates against the assembly based values from the JGI/IMG database resulted in an even better correlation ($r=0.90$, Figure S5 C) than that observed for rrnDB extrapolated RRN versus read-based RRN ($r= 0.59$; Figure S5 B). 16s rRNA genes contribute typically < 2% sequence length to the total genome size and 16s rRNA gene specific assembly errors will therefore have limited impact on the total genome size obtained after assembly. We concluded from the correlations displayed in Figures S4 and S5 that rrnDB extrapolated values were more reliable then RRN values from the JGI/IMG database. However, also rrnDB derived RRN estimates were likely impacted by biases due to the assembly process. In contrast, assembly related biases seemed to be less relevant for total genome size estimates and accordingly also the remaining genomic traits estimated from data provided via the JGI/IMG database. For the downstream trait-trait covariation and correlation analyses we used rrnDB

estimated RRN values, while all other trait data were obtained from the JGI/IMG database as detailed above.

Phylogenetic assignment of reference genomes All genomes sequence data used in this study (Table S1) were annotated via the GTDB-Tk software (Chaumeil et al., 2020) to obtain GTDB taxonomy annotations (Parks et al., 2022). Genomes that could not be unambiguously assigned to a single species were subsequently binned at the species level based on their average nucleotide identity (ANI, cutoff level: ANI>94%) using reciprocal classifications via the FastANI software (Jain et al., 2018).

Principal component analyses Principal component analyses (PCAs) were performed to visualize correlation structures among genomic traits. We have chosen to display the CUB parameter F (Vieira-Silva and Rocha, 2010) instead of generation time estimations that can be delineated from the CUB, because F is unambiguously defined for all genomes. In contrast, generation time estimations have been suggested to be inaccurate for genomes with large CUB values (Weissman et al., 2021). To reduce the phylogenetic redundancy of the dataset, trait values were averaged at the species level. The species level values for %HGT were log(x+0.001) transformed because this improved their fit to the normal distribution. The trait values were centered and scaled prior to all PCAs.

In the case of habitat specific analyses, three habitat types were determined via text search from the habitat information available via the JGI/IMG database (Supplementary Table S1; ignore.case=TRUE): all genomes containing the strings 'soil' or 'rhizosphere' in the habitat description where classified as originating from soil habitats; all genomes containing the strings 'aquatic' or 'marine' or 'water' where classified as originating from aquatic habitats; all genomes containing the strings 'oral' or 'stomach' or 'gut' or 'intestinal' or 'feces' were classified as originating from the intestinal tract. The remaining genomes were not further classified. Habitats were defined at the genome level and habitat assignment was not consistent for all genomes affiliating with the same species. Genomes affiliating with the same species that were not assigned to a single habitat type were therefore considered separately.

Correlation structures among genomic traits that were visualized via PCA biplots (Figure 3, Figure 6) were tested for their significance using permutation based statistics provided in the PCAtest R package (100 permutations, Camargo, 2022). The first and the second principal components (PC1, PC2) of all PCAs from this study contributed together to > 57% of total variability and were significant, while none of the remaining principal showed a significant contribution to the variability of genomic traits (Figure S2).

Pairwise correlations Global pairwise relationships among the genomic traits included in the PCAs were displayed in scatterplots (Figure 4). Partial correlation analyses (Spearman rank correlation) were performed for partial datasets, including genomes <4 Mbp, genomes or > 5 Mbp as well as genomes assigned to soil, aquatic or digestive tract environments (Table 2). Because it was not possible to obtain normal distributed trait values in all cases correlation strengths were determined using the Spearman rank-order correlations.

Phylogenetic signals We assessed phylogenetic signals of all genomic traits using Blomberg's K statistics as well as Pagel's Lamda statistics (Pagel, 1999; Blomberg et al., 2003) as implemented

in the phytools R package. We furthermore computed phylogenetic Mantel correlograms (200 permutations) similar as detailed elsewhere (Diniz-Filho et al., 2010; Dini-Andreote et al., 2015) to test for significant positive correlations between phylogenetic distances and trait distances at the following phylogenetic distance classes: 0-0.25 / 0.25-0.5 / 0.5-0.75 / 0.75-1 / 1-1.5 / 1.5-2 / 2-2.5/ 2.5-3. This method allowed to detect the phylogenetic levels at which phylogenetic signals are apparent. For the calculation of Mantel correlograms, the dataset was reduced to 10000 randomly selected genomes in order to reduce computation time and memory demand. The Mantel correlations were tested via the Spearman rank-order correlation as it was not possible to obtain normal distributed trait values in all cases.

Except for the trait RRN, the phylogeny of the prokaryotic PICRUSt2 default phylogenetic tree (Douglas et a. 2020: pro_ref.tre) was used to infer phylogenetic signals of the remaining genomic traits. In the case of RRN, our analyses indicated that values given in the JGI/IMG database may be affected by biases that were less apparent in the rrnDB database (Figure S4, S5). To infer phylogenetic signals for RRN we therefore used the trait table available via rrnDB database and a phylogenetic tree that was computed from the corresponding 16s rRNA gene sequence data. For this purpose 16s rRNA sequences were aligned after the removal of duplicates and using the MUSCLE software (Edgar, 2004). A phylogenetic tree was computed using the FastTree software (Price et al., 2010) and the GTR+Γ substitution model.

References

Biers, E. J., Sun, S. L., and Howard, E. C. (2009). Prokaryotic genomes and diversity in surface ocean waters:interrogating the Global Ocean Sampling metagenome. *Applied and Environmental Microbiology* 75, 2221–2229.

Blomberg, S. P., Garland, T., and Ives, A. R. (2003). Testing for phylogenetic signal in comparative data: Behavioral traits are more labile. *Evolution* 57, 717–745. doi: 10.1111/j.0014-3820.2003.tb00285.x.

Camargo, A. (2022). PCAtest: testing the statistical significance of Principal Component Analysis in R. *PeerJ* 10, e12967. doi: 10.7717/peerj.12967.

Chaumeil, P.-A., Mussig, A. J., Hugenholtz, P., and Parks, D. H. (2020). GTDB-Tk: a toolkit to classify genomes with the Genome Taxonomy Database. *Bioinformatics* 36, 1925–1927. doi: 10.1093/bioinformatics/btz848.

Chen, I.-M. A., Chu, K., Palaniappan, K., Ratner, A., Huang, J., Huntemann, M., et al. (2021). The IMG/M data management and analysis system v.6.0: new tools and advanced capabilities. *Nucleic Acids Research* 49, D751–D763. doi: 10.1093/nar/gkaa939.

Dini-Andreote, F., Stegen, J. C., Elsas, J. D. van, and Salles, J. F. (2015). Disentangling mechanisms that mediate the balance between stochastic and deterministic processes in microbial succession. *PNAS* 112, E1326–E1332. doi: 10.1073/pnas.1414261112.

Diniz-Filho, J. A. F., Terribile, L. C., Cruz, M. J. R. da, and Vieira, L. C. G. (2010). Hidden patterns of phylogenetic non-stationarity overwhelm comparative analyses of niche conservatism

and divergence. *Global Ecology and Biogeography* 19, 916–926. doi: https://doi.org/10.1111/j.1466-8238.2010.00562.x.

Douglas, G. M., Maffei, V. J., Zaneveld, J. R., Yurgel, S. N., Brown, J. R., Taylor, C. M., et al. (2020). PICRUSt2 for prediction of metagenome functions. *Nature Biotechnology* 38, 685–688. doi: 10.1038/s41587-020-0548-6.

Edgar, R. C. (2004). MUSCLE: a multiple sequence alignment method with reduced time and space complexity. *BMC Bioinformatics* 5, 1–19. doi: 10.1186/1471-2105-5-113.

Jain, C., Rodriguez-R, L. M., Phillippy, A. M., Konstantinidis, K. T., and Aluru, S. (2018). High throughput ANI analysis of 90K prokaryotic genomes reveals clear species boundaries. *Nat Commun* 9, 5114. doi: 10.1038/s41467-018-07641-9.

Kopylova, E., Noe, L., and Touzet, H. (2012). SortMeRNA: fast and accurate filtering of ribosomal RNAs in metatranscriptomic data. *Bioinformatics* 28, 3211–3217. doi: 10.1093/bioinformatics/bts611.

Mistry, J., Chuguransky, S., Williams, L., Qureshi, M., Salazar, G. A., Sonnhammer, E. L. L., et al. (2021). Pfam: The protein families database in 2021. *Nucleic Acids Research* 49, D412–D419. doi: 10.1093/nar/gkaa913.

Mukherjee, S., Stamatis, D., Bertsch, J., Ovchinnikova, G., Sundaramurthi, J. C., Lee, J., et al. (2021). Genomes OnLine Database (GOLD) v.8: overview and updates. *Nucleic Acids Research* 49, D723–D733. doi: 10.1093/nar/gkaa983.

Nayfach, S., and Pollard, K. S. (2015). Average genome size estimation improves comparative metagenomics and sheds light on the functional ecology of the human microbiome. *Genome Biol.* 16, 51. doi: 10.1186/s13059-015-0611-7.

Pagel, M. (1999). Inferring the historical patterns of biological evolution. *Nature* 401, 877–884. doi: 10.1038/44766.

Parks, D. H., Chuvochina, M., Rinke, C., Mussig, A. J., Chaumeil, P.-A., and Hugenholtz, P. (2022). GTDB: an ongoing census of bacterial and archaeal diversity through a phylogenetically consistent, rank normalized and complete genome-based taxonomy. *Nucleic Acids Res* 50, D785–D794. doi: 10.1093/nar/gkab776.

Price, M. N., Dehal, P. S., and Arkin, A. P. (2010). FastTree 2-Approximately Maximum-Likelihood Trees for Large Alignments. *PLoS One* 5. doi: 10.1371/journal.pone.0009490.

Roux, S., Enault, F., Hurwitz, B. L., and Sullivan, M. B. (2015). VirSorter: mining viral signal from microbial genomic data. *PeerJ* 3, e985. doi: 10.7717/peerj.985.

Stoddard, S. F., Smith, B. J., Hein, R., Roller, B. R. K., and Schmidt, T. M. (2015). rrnDB: improved tools for interpreting rRNA gene abundance in bacteria and archaea and a new foundation for future development. *Nucleic Acids Res.* 43, D593–D598. doi: 10.1093/nar/gku1201.

Vieira-Silva, S., and Rocha, E. P. C. (2010). The Systemic Imprint of Growth and Its Uses in Ecological (Meta) Genomics. *PLoS Genet.* 6, e1000808. doi: 10.1371/journal.pgen.1000808.

Weissman, J. L., Hou, S., and Fuhrman, J. A. (2021). Estimating maximal microbial growth rates from cultures, metagenomes, and single cells via codon usage patterns. *PNAS* 118. doi: 10.1073/pnas.2016810118.

Wilson, D., Charoensawan, V., Kummerfeld, S. K., and Teichmann, S. A. (2008). DBDtaxonomically broad transcription factor predictions: new content and functionality. *Nucleic Acids Res.* 36, D88–D92. doi: 10.1093/nar/gkm964.
