# Supplementary figures and images for: Trait-trait relationships and tradeoffs vary with genome size in prokaryotes
Source: Front Microbiol. 2022 Oct 21;13:985216. doi: 10.3389/fmicb.2022.985216 (PMC9634001; doi:10.3389/fmicb.2022.985216)

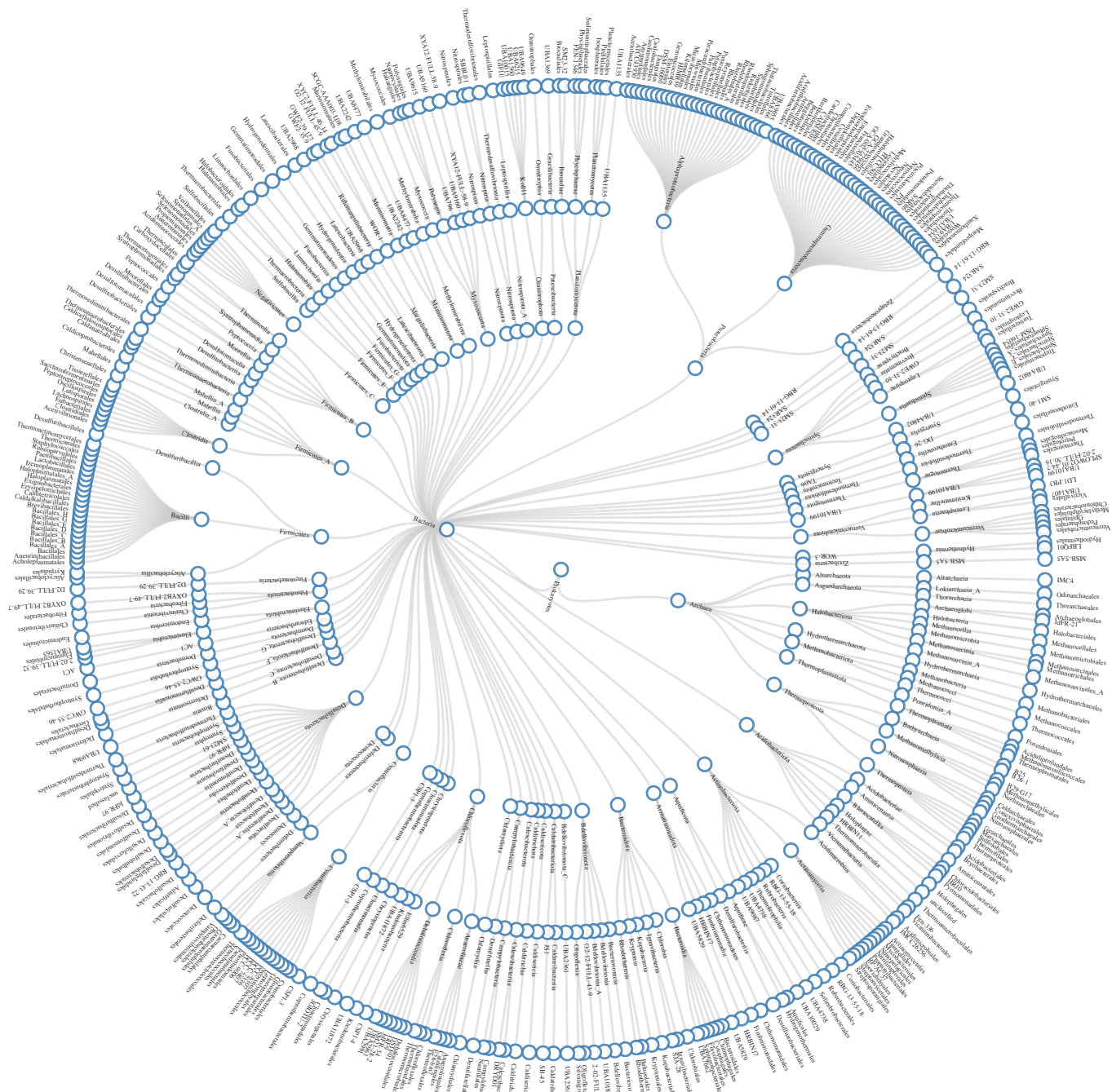

Supplement: Supplementary file 3 [file Image_1.pdf]
